# Supplementary material for: Novel Markers of the Metabolic Impact of Exogenous Retinoic Acid with A Focus on Acylcarnitines and Amino Acids
Source: Int J Mol Sci. 2019 Jul 25;20(15):3640. doi: 10.3390/ijms20153640 (PMC6696161; doi:10.3390/ijms20153640)
Supplement: Supplementary file 1 [file ijms-20-03640-s001.pdf]

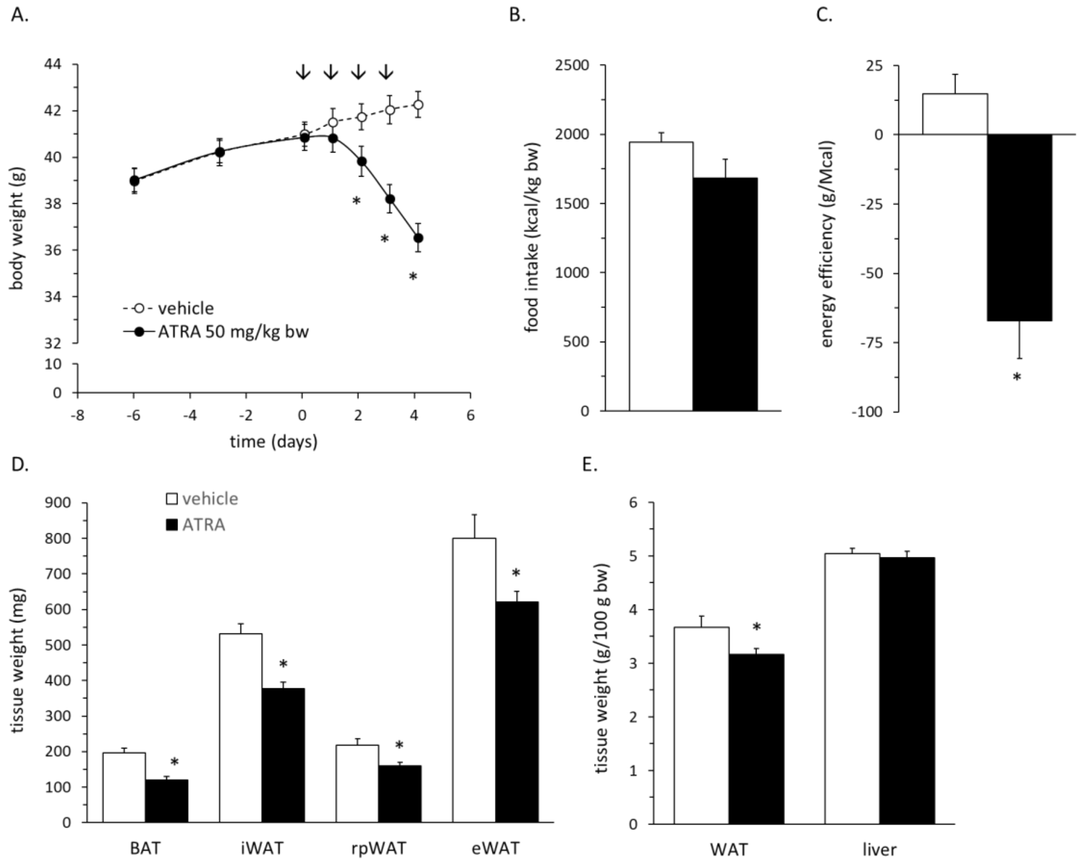

Supplementary Figure 1. Body weight evolution (A), food intake (B), and energy efficiency, as g body weight gained per kg diet fed, (C) during ATRA treatment (from day 0 to day 4). Adipose tissue depots weights (D) and adiposity and liver index, as sum of weights of white adipose tissue depots or liver per 100 g of body weight (E) at sacrifice. NMRI mice received one daily subcutaneous injection of ATRA at a dose of 50 mg/kg body weight or the vehicle (olive oil) during the 4 days before they were sacrificed. Data are the means ± SEM of 11 mice per group (in A,D and E) or of 4 cages per group (in B and C). * Indicates significant difference between ATRA and the control group (P <0.05, *two-tailed Student's t test*).

Supplementary Table 1. Total variance and principal components loadings for the first five principal components (PC).

	Principal components				
	**PC1**	**PC2**	**PC3**	**PC4**	**PC5**
**Portion of variance** (%)	22.65	18.20	9.93	5.98	5.46
**Cumulative variance** (%)	22.65	40.85	50.78	56.76	62.22
	**Principal components loadings**				
**Variable**	**PC1**	**PC2**	**PC3**	**PC4**	**PC5**
**Plasma acylcarnitines** (UA/volumen, %)					
Carnitine	0.507	**0.646**	-0.223	-0.015	0.244
C2:0	-0.117	**0.894**	-0.069	-0.045	0.137
C3:0	0.583	0.576	-0.081	-0.137	-0.278
C4:0	0.411	0.316	0.164	-0.117	**-0.401**
C4DC	0.421	0.439	0.171	-0.315	0.292
C4OH	-0.557	**0.625**	0.282	-0.173	-0.045
C5:0	-0.187	0.505	**0.616**	0.003	-0.031
C5OH	0.477	0.400	0.174	-0.369	0.181
C6:0	-0.111	0.559	0.055	-0.161	-0.341
C8:0	-0.570	**0.609**	0.042	0.109	-0.097
C10:0	-0.165	-0.200	-0.053	0.016	0.066
C12:0	**-0.768**	0.396	0.407	0.065	-0.050
C12:1	**-0.757**	0.499	0.266	0.063	0.121
C14:0	-0.718	0.228	0.457	0.123	-0.082
C14:1	**-0.782**	0.449	0.309	0.178	-0.027
C16:0	-0.693	0.263	0.412	-0.079	-0.050
C16:1	**-0.807**	0.374	0.396	0.060	0.015
C16DC	-0.402	**0.606**	0.060	-0.087	-0.021
C18:0	-0.729	0.313	0.239	-0.150	0.040
C18:1	**-0.814**	0.419	0.353	0.038	0.067
C18:2	**-0.785**	0.483	0.314	0.073	-0.053

C20:4	-0.711	0.401	**0.508**	0.020	0.114
**Plasma amino acids** (UA/volumen, %)					
Alanine	**0.768**	0.487	0.083	-0.267	-0.003
Arginine	**0.836**	0.369	0.206	-0.037	-0.063
Asparagine	0.625	0.568	0.349	0.028	-0.103
Aspartate	0.329	-0.013	**0.681**	-0.155	-0.015
Citrulline	0.716	-0.166	0.438	-0.015	0.086
Creatine	0.341	0.486	0.131	**-0.462**	0.293
Creatinine	0.327	0.125	0.293	-0.304	0.132
Cystine	0.511	0.587	-0.015	-0.305	0.180
Glutamate	0.465	0.199	**0.647**	-0.145	-0.006
Glutamine	0.668	**0.650**	0.173	0.167	0.103
Glycine	0.390	0.552	0.012	0.092	-0.290
Histidine	0.668	0.586	0.200	0.167	0.208
Homocysteine	**0.879**	0.188	0.285	0.086	-0.098
Homocystine	-0.628	0.171	0.258	0.176	**-0.502**
Leucine - Ile	0.618	0.341	**0.533**	0.030	-0.107
Methionine	**0.799**	0.255	0.247	0.045	-0.079
Ornithine	**0.797**	0.113	**0.505**	0.048	-0.134
Phenylalanine	0.697	0.485	0.292	0.142	-0.076
Proline	**0.862**	0.159	0.263	0.036	-0.216
Serine	0.637	0.329	0.448	-0.170	-0.016
Serotonin	-0.207	-0.232	0.316	-0.168	-0.254
Taurine	0.386	**0.597**	-0.116	0.127	-0.007
Threonine	**0.850**	0.304	0.168	0.004	-0.076
Tryptophan	0.510	0.197	0.123	**0.382**	-0.175
Tyrosine	**0.880**	0.315	0.208	0.165	0.044
Valine	0.743	0.288	0.438	0.016	-0.156
**Plasma lipids and intermediate metabolites** (UA/volumen, %)					
Cholesterol	0.336	0.228	-0.199	**0.399**	-0.286

Palmitic acid	-0.572	-0.252	**0.515**	0.190	-0.155
Oleic acid	-0.234	0.032	**0.574**	0.098	0.229
Arachidonic acid	-0.558	0.463	0.289	0.121	0.114
DHA	-0.329	**0.715**	0.147	0.090	0.094
EPA	-0.516	0.392	0.430	-0.013	0.329
Hexose	0.683	0.429	-0.151	0.198	0.288
HexoseP	0.137	-0.284	**0.700**	-0.225	0.274
Lactate	0.225	0.422	-0.121	0.186	0.169
Citrate - isocitrate	0.593	0.403	-0.091	0.234	0.278
Malate	0.367	0.564	-0.150	0.126	0.077
AMP	-0.385	-0.053	0.276	-0.225	**-0.388**
Nicotinamide	-0.302	-0.037	**0.625**	-0.316	0.179
Choline	0.325	0.315	**0.729**	0.057	0.102
Urea	**0.782**	0.120	0.200	-0.094	-0.094
**Energy efficiency** (g bw gain/kg diet fed)	0.510	**-0.730**	0.223	0.056	-0.126
**Brown adipose tissue (BAT)**					
Tissue weight (mg)	0.118	**-0.793**	0.248	0.026	**0.394**
Atp5f1 mRNA (%)	0.102	0.153	-0.023	0.325	**-0.435**
Cox8b mRNA (%)	0.103	0.588	-0.407	0.056	**-0.393**
Ppara mRNA (%)	-0.409	0.411	0.297	**0.447**	-0.155
Pparg mRNA (%)	-0.149	0.329	0.325	**0.484**	-0.259
Rarb mRNA/*Rxrg* mRNA (%)	0.356	0.245	0.040	-0.191	0.208
Tmem26 mRNA (%)	-0.017	-0.099	0.199	**0.469**	-0.203
Ucp1 mRNA (%)	-0.215	**0.761**	-0.370	0.037	-0.172
**Inguinal white adipose tissue (iWAT)**					
Tissue weight (mg)	0.161	**-0.712**	0.296	0.172	0.307
Atp5f1 mRNA (%)	-0.447	-0.181	0.046	-0.173	0.151
Tnfrsf9 (Cd137) mRNA (%)	-0.737	0.285	0.196	-0.161	-0.014
Cox8b mRNA (%)	-0.176	-0.266	0.229	-0.059	-0.025
Hoxc9 mRNA (%)	0.314	-0.172	**-0.491**	**0.676**	0.297

Ppargc1b mRNA (%)	-0.089	0.270	0.005	0.031	0.204
Ppara mRNA (%)	-0.101	0.407	-0.002	**0.500**	0.300
Pparg mRNA (%)	0.314	0.009	0.199	-0.080	0.129
Rarb mRNA/*Rxrg* mRNA (%)	-0.499	0.495	-0.397	-0.332	-0.021
Slc27a1 mRNA (%)	-0.090	0.509	0.098	0.163	-0.262
Tbx1 mRNA (%)	0.248	0.044	-0.316	0.344	0.225
Tfb2m mRNA (%)	-0.721	0.254	0.249	-0.042	0.272
Tmem26 mRNA (%)	-0.007	-0.325	0.056	**0.598**	-0.231
Ucp1 mRNA (%)	-0.228	0.364	-0.060	**0.580**	0.028
Yy1 mRNA (%)	0.263	-0.063	-0.375	**0.682**	**0.388**
**Retroperitoneal white adipose tissue (rpWAT)**					
Tissue weight (mg)	0.182	-0.480	**0.524**	0.079	**0.467**
Atp5f1 mRNA (%)	0.230	0.442	**-0.564**	-0.239	-0.219
Tnfrsf9 (Cd137) mRNA (%)	0.187	0.401	-0.016	**-0.376**	0.068
Cox8b mRNA (%)	0.326	0.527	-0.270	-0.217	-0.331
Hoxc9 mRNA (%)	0.128	0.533	-0.241	-0.255	-0.315
Ppargc1b mRNA (%)	-0.238	0.203	-0.129	-0.176	**-0.457**
Ppara mRNA (%)	-0.343	**0.621**	-0.121	0.058	-0.148
Pparg mRNA (%)	0.016	**-0.655**	**-0.482**	-0.081	-0.215
Rarb mRNA/*Rxrg* mRNA (%)	-0.042	0.432	-0.422	-0.102	**0.439**
Slc27a1 mRNA (%)	-0.096	0.594	-0.472	0.137	-0.062
Tbx1 mRNA (%)	0.193	0.002	-0.140	-0.114	0.075
Tfb2m mRNA (%)	-0.041	0.392	-0.335	-0.016	-0.361
Tmem26 mRNA (%)	0.035	**-0.684**	0.081	-0.129	-0.098
Ucp1 mRNA (%)	0.179	0.473	-0.238	-0.061	0.084
Yy1 mRNA (%)	-0.295	0.579	-0.082	0.202	-0.240
**Epididymal white adipose tissue (eWAT)**					
Tissue weight (mg)	0.173	-0.332	0.472	0.243	**0.380**
Atp5f1 mRNA (%)	-0.246	0.304	0.029	-0.267	**0.464**
Tnfrsf9 (Cd137) mRNA (%)	0.086	0.484	-0.291	0.195	**0.560**

Cox8b mRNA (%)	0.190	0.334	-0.346	0.062	0.224
Hoxc9 mRNA (%)	-0.284	-0.042	-0.102	**-0.533**	-0.223
Ppargc1b mRNA (%)	-0.054	0.379	-0.186	0.258	**0.468**
Ppara mRNA (%)	0.181	0.019	-0.291	**-0.598**	-0.147
Pparg mRNA (%)	0.057	-0.359	0.201	0.362	0.022
Rarb mRNA/*Rxrg* mRNA (%)	-0.476	0.383	-0.047	**0.552**	-0.072
Slc27a1 mRNA (%)	-0.335	0.552	-0.274	0.087	0.325
Tbx1 mRNA (%)	-0.066	0.553	-0.099	0.188	-0.053
Tfb2m mRNA (%)	-0.551	0.486	-0.285	-0.185	0.365
Tmem26 mRNA (%)	0.437	-0.324	0.143	0.115	-0.132
Ucp1 mRNA (%)	0.087	0.567	-0.379	-0.277	**0.452**
Yy1 mRNA (%)	-0.480	0.082	-0.235	-0.328	0.349

The 15 most significant loadings are indicated in bold. Data of 11 animals per group were included in the PC analysis.

Supplementary Table 2. Correlations between plasma metabolites better discriminating between control and all-trans retinoic acid-treated mice according to PCA (in AU/volume, %) and selected variables related to thermogenic capacity in adipose tissues.

		Carnitine	C2:0	C4OH	C8:0	C16DC	DHA	Taurine	Glutamine
**Energy efficiency** (g bw gain/kg diet fed)	ρ	-0.270	-0.759**	-0.521*	-0.490*	-0.727**	-0.665**	-0.053	-0.049
	P	0.224	0.000	0.013	0.021	0.000	0.001	0.816	0.828
	n	22	22	22	22	22	22	22	22
**Brown adipose tissue**									
Tissue weight (mg)	ρ	-0.365	-0.744**	-0.470*	-0.462*	-0.591**	-0.642**	-0.282	-0.232
	P	0.113	0.000	0.036	0.040	0.006	0.002	0.228	0.326
	n	20	20	20	20	20	20	20	20
Ucp1 mRNA (%)	ρ	0.335	0.783**	0.474*	0.618**	0.513*	0.658**	0.219	0.184
	P	0.128	0.000	0.026	0.002	0.015	0.001	0.327	0.414
	n	22	22	22	22	22	22	22	22
Ppara mRNA (%)	ρ	0.346	0.484*	0.469*	0.467	0.761**	0.781**	0.336	0.240
	P	0.160	0.042	0.050	0.050	0.000	0.000	0.173	0.336
	n	18	18	18	18	18	18	18	18
**Subcutaneous white adipose tissue (inguinal)**									
Tissue weight (mg)	ρ	-0.390	-0.698**	-0.597**	-0.405	-0.551*	-0.542*	-0.136	-0.109
	P	0.089	0.001	0.005	0.076	0.012	0.014	0.567	0.647
	n	20	20	20	20	20	20	20	20
Ucp1 mRNA (%)	ρ	0.203	0.215	-0.145	0.192	0.129	0.183	0.223	0.147
	P	0.391	0.363	0.541	0.416	0.587	0.439	0.345	0.535
	n	20	20	20	20	20	20	20	20
Ppara mRNA (%)	ρ	0.258	0.435	0.357	0.451	0.247	0.461*	0.255	0.246
	P	0.286	0.063	0.133	0.053	0.307	0.047	0.293	0.311
	n	19	19	19	19	19	19	19	19
**Visceral white adipose tissues (retroperitoneal & epididymal)**									
Tissue weight (mg)	ρ	-0.109	-0.196	-0.171	-0.080	-0.247	-0.176	0.037	0.020
	P	0.502	0.225	0.290	0.625	0.125	0.278	0.821	0.901

	n	40	40	40	40	40	40	40	40
Ucp1 mRNA (%)	ρ	0.487**	0.552**	0.443**	0.102	0.434**	0.352*	0.333*	0.376*
	P	0.001	0.000	0.004	0.530	0.005	0.026	0.036	00.017
	n	40	40	40	40	40	40	40	40
Ppara mRNA (%)	ρ	0.273	0.164	0.140	0.138	0.222	0.171	0.157	0.141
	P	0.088	0.312	0.390	0.395	0.169	0.292	0.334	0.386
	n	40	40	40	40	40	40	40	40

Shown in the Table are the Spearman rank-order correlation coefficient (ρ), the statistical significance (P), and the number of animals (n). ** indicates that the correlation is significant at the observed P-value of 0.01 (bilateral) and * indicates that the correlation is significant at the observed P-value of 0.05 (bilateral) after Benjamini-Hochberg correction. AU, arbitrary units.
